# P-1133. Salvage of the infected Nuss bar: A protocol and institutional experience

**DOI:** 10.1093/ofid/ofaf695.1327

**Published:** 2026-01-11

**Authors:** Christopher Clinker, Jack H Scaife, Nicolas M Cordoba, Katie Russell, Trahern Wallace. Jones

**Affiliations:** University of Utah School of Medicine, SALT LAKE CITY, Utah; University of Utah Department of Surgery, Salt Lake City, Utah; University of Utah, Johnson city, New York; University of Utah, Johnson city, New York; University of Utah, Johnson city, New York

## Abstract

**Background:**

Surgical implant infections frequently lead to early removal of hardware that may introduce additional complications to a patient’s care. Nuss bar implants are placed via minimally-invasive surgery to correct pectus excavatum. Prior literature has described that Nuss bar infections are rare and amenable to medical salvage therapy with long-term antibiotics. However, there are no published guidelines or standardized protocols to assist providers in managing these infections.

**Methods:**

This study describes the implementation of a Nuss bar infection salvage protocol. This protocol was developed around six adolescent patients who underwent minimally-invasive Nuss bar implantations for pectus excavatum and suffered subsequent device-related infection between October 2021 and October 2024. A detailed chart review was completed.

**Results:**

Six patients with infection after Nuss procedure were identified. In total there were 221 Nuss procedures for an infection rate of 2.7%. Infection was determined both clinically and intraoperatively and confirmed with positive culture results. Infections presented from 9 to 61 days following initial placement, with a median of 26 days. Five out of six infections were associated with growth of *Staphylococcus aureus* from intraoperative cultures. Four of these were methicillin-sensitive, while the fifth was methicillin-resistant. The sixth patient’s intraoperative cultures were positive for *Pseudomonas aeruginosa*. Based on the protocol that was developed, all patients underwent 2 - 4 operative washout and debridement procedures with wound vac therapy between procedures. They were initially treated with broad spectrum antibiotics and then culture-directed antibiotic therapy for a total of 12 weeks. Five of six patients were transitioned to early oral antibiotics at discharge, and one required 12 weeks of intravenous antibiotics due to severe oral aversion. All patients retained their hardware and had no evidence of relapse of infection following completion of their salvage therapy.

**Conclusion:**

Nuss bar infections are rare. Experience has demonstrated successful salvage with a standardized protocol incorporating 12 weeks of directed antibiotic therapy, serial washouts, and close follow-up, with no relapses of infection.

**Disclosures:**

Nicolas M. Cordoba, MD, MBA, Striker, Johnson and Johnson, Pfizer, and others throughout ETF: Stocks/Bonds (Public Company)

